# Genetic basis of nectar guide trichome variation between bumblebee- and self-pollinated monkeyflowers (*Mimulus*): role of the *MIXTA*-like gene *GUIDELESS*

**DOI:** 10.1186/s12870-024-04736-y

**Published:** 2024-01-23

**Authors:** Hongfei Chen, Yao-Wu Yuan

**Affiliations:** 1 Department of Ecology and Evolutionary Biology, University of Connecticut, Storrs, CT 06269 USA; 2 Institute for Systems Genomics, University of Connecticut, Storrs, CT 06269 USA

**Keywords:** *Mimulus lewisii*, *Mimulus parishii*, Pollination syndrome, Near-isogenic line (NIL), *R2R3-MYB*

## Abstract

Nectar guide trichomes play crucial ecological roles in bee-pollinated flowers, as they serve as footholds and guides for foraging bees to access the floral rewards. However, the genetic basis of natural variation in nectar guide trichomes among species remains poorly understood. In this study, we performed genetic analysis of nectar guide trichome variation between two closely related monkeyflower (*Mimulus*) species, the bumblebee-pollinated *Mimulus lewisii* and self-pollinated *M. parishii*. We demonstrate that a *MIXTA*-like *R2R3*-*MYB* gene, *GUIDELESS*, is a major contributor to the nectar guide trichome length variation between the two species. The short-haired *M. parishii* carries a recessive allele due to non-synonymous substitutions in a highly conserved motif among MIXTA-like MYB proteins. Furthermore, our results suggest that besides *GUIDELESS*, additional loci encoding repressors of trichome elongation also contribute to the transition from bumblebee-pollination to selfing. Taken together, these results suggest that during a pollination syndrome switch, changes in seemingly complex traits such as nectar guide trichomes could have a relatively simple genetic basis, involving just a few genes of large effects.

**Supplementary Information:**

The online version contains supplementary material available at 10.1186/s12870-024-04736-y.

## Introduction

Plant-pollinator interactions are ubiquitous and are estimated to involve ~ 170,000 plant and ~ 200,000 animal species in nature [1]. Pollinator-mediated selection is considered a major driver for floral trait diversification and angiosperm speciation [2–6]. Flowers pollinated by a particular type of pollinator often share certain commonalities. For example, many bumblebee-pollinated flowers, especially bilaterally symmetric flowers, contain a ventral petal with variously arranged trichomes and contrasting coloration compared to the other petals, which together serve as nectar guides for the pollinators [7–13]. Nectar guide trichomes are used as footholds for foraging bees to enter flowers [10, 14] and may trap pollen from insects for subsequent pollen transfer to the stigma when pollinators are scarce, thereby increasing reproductive success [9]. Despite their obvious ecological importance, very little is known about the genetic basis of natural variation in nectar guide trichomes among species.

The basic genetic network controlling trichome development, especially in vegetative tissues, has been extensively studied in several plant model systems. In *Arabidopsis*, a regulatory complex composed of transcription factors belonging to three families positively regulates trichome initiation, including the R2R3-MYB proteins GLABROUS1 (GL1), AtMYB23 [15], and AtMYB82 [15–17], the basic helix-loop-helix (bHLH) proteins GLABRA3 (GL3), ENHANCER OF GLABRA3 (EGL3), TRANSPARENT TESTA 8 (TT8), and AtMYC-1 [18–21], and the WD40 repeat (WDR) protein TRANSPARENT TESTA GLABRA1 (TTG1) [22]. Transcription factors belonging to the same family are at least partially redundant in function. This MYB-bHLH-WDR (MBW) regulatory complex activates the downstream gene *GLABRA2* (*GL2*), which encodes a homeodomain leucine-zipper (HD-ZIP) protein that determines trichome cell fate in shoots [23, 24]. Additionally, a group of C2H2 zinc finger transcription factors, including GLABROUS INFLORESCENCE STEMS (GIS), GIS2, ZINC FINGER PROTEIN5 (ZFP5), ZFP6, and ZFP8 [25–28], function as positive regulators of trichome initiation upstream of the MBW regulatory complex. In addition to these activators, a group of single repeat R3-MYBs, including CAPRICE (CPC) [29], TRIPTYCHON (TRY) [30], ENHANCER OF TRY AND CPC1 (ETC1) [31], ETC2 [32], and TRICHOMELESS1 (TCL1) were found to function redundantly as negative regulators of trichome determination by competing with the R2R3-MYBs for bHLH binding [33, 34]. Although most of these genes were identified through mutant analyses, some of them are also known to cause trichome variation among natural accessions of *Arabidopsis*. For example, a single amino acid substitution in ETC2 is responsible for reduced trichome density on *Arabidopsis* leaves in natural populations [35]. *TCL1* and *TRY* were also identified as causal genes underlying diversity of trichome patterning in fruits or pedicels in natural *Arabidopsis* populations [36].

However, recent studies in many crop and medicinal plants, including maize, cotton, tomato, cucumber, melon, and *Artemisia annua*, suggest that the regulation of trichome development by the MBW complex uncovered in *Arabidopsis* seems more the exception than the rule. In most other plant species, the core module that positively regulates trichome initiation and elongation contains the MIXTA-like R2R3-MYB belonging to a different subgroup than GL1 [37–47] and class IV HD-ZIP transcription factors [48–53]. Other positive regulators of trichome development that genetically or biochemically interact with the MIXTA-like R2R3-MYB or/and HD-ZIP IV proteins include C2H2 zinc-finger proteins (e.g., H in tomato and Tu in cucumber) [54, 55] and MYC1-like bHLH proteins (e.g., SlMYC1 in tomato) [56], whereas negative regulators include B-type cyclin-like and Jasmonate ZIM (JAZ) proteins [51, 56–58]. These remarkable progresses in the past decade have set the stage for dissecting the genetic basis underlying nectar guide trichome variation between species with different pollination guilds.

**Fig. 1:**
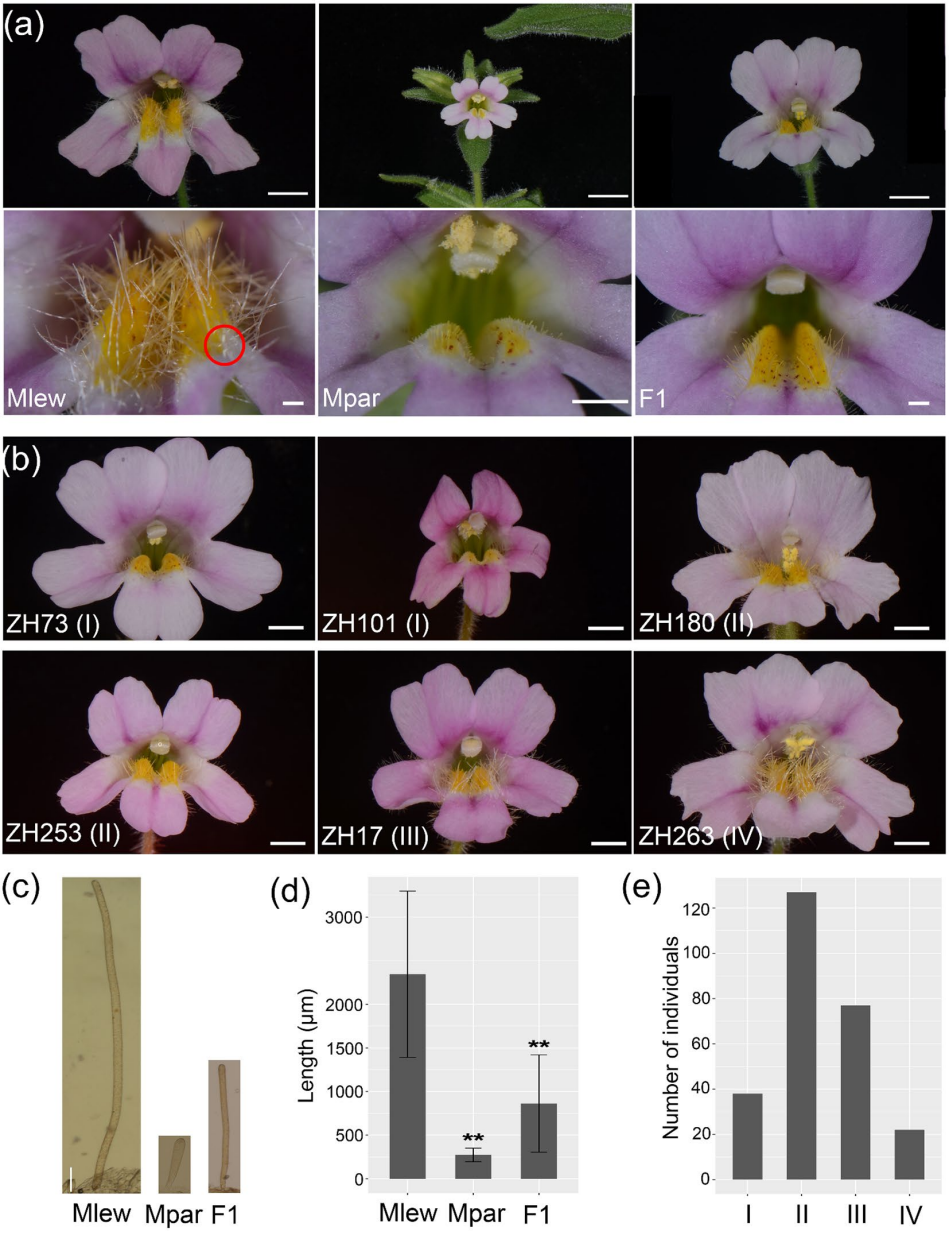
Phenotypes of nectar guide trichomes. (**a**) Front view of the whole corolla (upper) and enlarged view of the corolla throat of *M*. *lewisii* (Mlew, left), *M*. *parishii* (Mpar, middle), and their F1 hybrid (right). Scale bars, 5 mm (top row) and 1 mm (bottom row). The red circle marks the position where nectar guide trichome length were quantified. (**b**) Front view of F2 hybrids representing the four classes. Scale bars, 4 mm. (**c**) Light microscopy images of nectar guide trichomes of *M*. *lewisii*, *M*. *parishii* and F1 hybrids. Scale bars, 200 μm. (**d**) Quantification of nectar guide trichome length (*n* = 20 for each genotype). Error bars are 1 SD. Asterisks indicate differences from *M*. *lewisii* (** *p* < 0.01, student’s t test). (**e**) Frequency distribution of the four classes of F2 individuals

The monkeyflower species *Mimulus lewisii* and *M. parishii* are closely related [59, 60] but display different pollination modes. The bumblebee-pollinated *M. lewisii* bears large showy flowers with long nectar guide trichomes (Fig. 1a, left panel), whereas the self-pollinated *M. parishii* produces small flowers with very short nectar guide trichomes (Fig. 1a, middle panel). These species are easy to grow in the greenhouse, with short generation time and high fecundity, and are amenable to transgenic manipulation through *Agrobacterium*-mediated transformation [61, 62]. This study represents the first step towards a detailed dissection of the genetic basis underlying nectar guide trichome variation between the two species. Starting from an F2 population with a wide range of nectar guide trichome lengths (Fig. 1b), we constructed near-isogenic lines (NILs) in the *M. lewisii* genetic background to reduce the complexity of F2 phenotypes into Mendelian loci. Through bulked segregant analysis, fine-scale genetic mapping, and complementation crosses, we demonstrate that the *MIXTA*-like gene, *GUIDELESS* [46], is a major contributor to the nectar guide trichome variation between the two species.

## Materials and methods

### Plant materials and growth conditions

The *M*. *lewisii* inbred line LF10, the *guideless* mutant, and the *M. parishii* inbred line Mpar were described previously [46, 61, 62]. All plants were grown in the University of Connecticut EEB research greenhouses under natural light supplemented with sodium vapor lamps, ensuring a 16-hr day length with a light intensity of 110–160 µmol·m^− 2^·s^− 1^. Plants were fertilized 2–3 times per week.

### Quantification of nectar guide trichome length

Nectar guide tissues at the same site near the throat (marked by a red circle in Fig. 1a) of the corolla were cut into small pieces. The trichomes in these tissues were then imaged under a light microscope, and their lengths were measured using Zeiss ZEN 2.6 lite (blue edition) software.

### NIL construction

To construct NILs with loci controlling nectar guide trichome variation introgressed from *M. parishii* to *M*. *lewisii*, we selected an F2 individual with short trichomes, ZH101 (Fig. 1b), to backcross (BC) to *M*. *lewisii*, and then we selfed one BC_1_ individual that most closely resembled *M*. *lewisii*, including the long nectar guide trichomes. The short-haired phenotype reappeared in the selfing population (BC_1_S_1_). The same process was repeated for a second time. The short vs. long trichome segregated ~ 1:3 in the BC_2_S_1_ population. The short-haired individuals from the BC_2_S_1_ population were then subjected to bulked segregant analysis by genome sequencing to identify the causal locus (see the section below). One of the short-haired BC_2_S_1_ individuals was backcrossed to *M*. *lewisii* again to clean up the residual *M. parishii* DNA, and one short-haired BC_3_S_1_ individual with only the causal locus introgressed from *M. parishii* was retained as the *G*^*P/P*^ NIL (named for the causal gene *GUIDELESS*; see Results).

### Bulked segregant analysis by deep sequencing

To determine which chromosome fragment (s) was introgressed from *M. parishii* to *M. lewisii* in the *G*^*P/P*^ NIL, we performed bulked segregant analysis, following [63]. Briefly, we pooled DNA samples from 39 short-haired individuals from the ZH101 BC_2_S_1_ population, with equal representation from each sample. A small-insert (350-bp) library was prepared for the pooled sample, and 150-bp paired-end reads were generated by Illumina NovaSeq at Novogene (Sacramento, CA), with ~ 80-fold genome coverage. The resulting short reads were mapped to the *M. lewisii* reference genome (http://mimubase.org/FTP/Genomes/LF10g_v2.0/) with CLC Genomics Workbench 7.0. After SNP calling, the number of homozygous SNPs in 20-kb bins was plotted in a bar graph.

### Expression analysis using RT-qPCR

Total RNA was extracted using the Spectrum Plant Total RNA Kit (TRN250, Sigma-Aldrich) and cDNA was synthesized from 500 ng of the DNaseI (Invitrogen) treated RNA using GoScript™ Reverse Transcription Mix (A2791, Promega), then diluted 10-fold before RT-qPCR. To normalize expression levels, the *M. lewisii*/*M. parishii* ortholog of *Arabidopsis* ubiquitin-conjugating enzyme gene (At5g25760), *MIUBC*, was used as reference gene following [62]. RT-qPCR was performed using iQ^TM^SYBR® Green Supermix (Bio-Rad) on a CFX96 Touch Real-Time PCR Detection System (Bio-Rad). The cDNA samples were amplified for 40 cycles of 95 °C for 15 s and 60 °C for 60 s. Amplification efficiencies for each primer pair were determined using critical threshold values obtained from a dilution series (1:4, 1:8, 1:16, 1:32) of pooled cDNAs. Relative expression of *GUIDELESS* was calculated using the formula (E_target_)^CP (ref)^/ (E^ref^) ^CP (target)^. The primers used for RT-(q)PCR are listed in Table S1.

## Results

### Variation in nectar guide trichome length between *M. lewisii* and *M. parishii*

Measurements using the microscope images showed that the nectar guide trichome length of *M*. *lewisii* is about 8 times that of *M*. *parishii* and about 3 times that of the F1 hybrid (Fig. 1c, d). We visually scored the nectar guide trichome lengths of 264 F2 individuals, and categorized them into four classes (examples shown in Fig. 1b). Class I (14.4%; 38/264) have very short trichomes resembling *M. parishii*. Class IV (8.3%; 22/264) showed long trichomes similar to *M. lewisii*. Class II (48.5%; 128/264) and class III (28.8%; 128/264) showed intermediate trichome lengths, with the former closer to *M. parishii* and the latter closer to *M. lewisii* (Fig. 1e). The frequency distribution of the F2 phenotypes suggests a non-monogenic basis of nectar guide trichome variation between the two species.

### Identification of *GUIDELESS* as a candidate gene underlying one of the causal loci

To reduce the variation in nectar guide trichome length displayed by the F2 population into mendelian loci, we took a near-isogenic line (NIL) approach. Specifically, we selected the F2 individual ZH101 (Fig. 1b) bearing short nectar guide trichomes, and backcrossed it to *M*. *lewisii*. Within the resulting BC_2_S_1_ population (two rounds of backcrossing and selfing; see Materials and Methods), approximately 3/4 (111/154) of the individuals had nectar guide trichomes similar to the wild type *M*. *lewisii* and ~ 1/4 (43/154) had shorter trichomes. We thus inferred that this short-haired NIL is homozygous for the recessive *M*. *parishii* allele at a single causal locus.

**Fig. 2:**
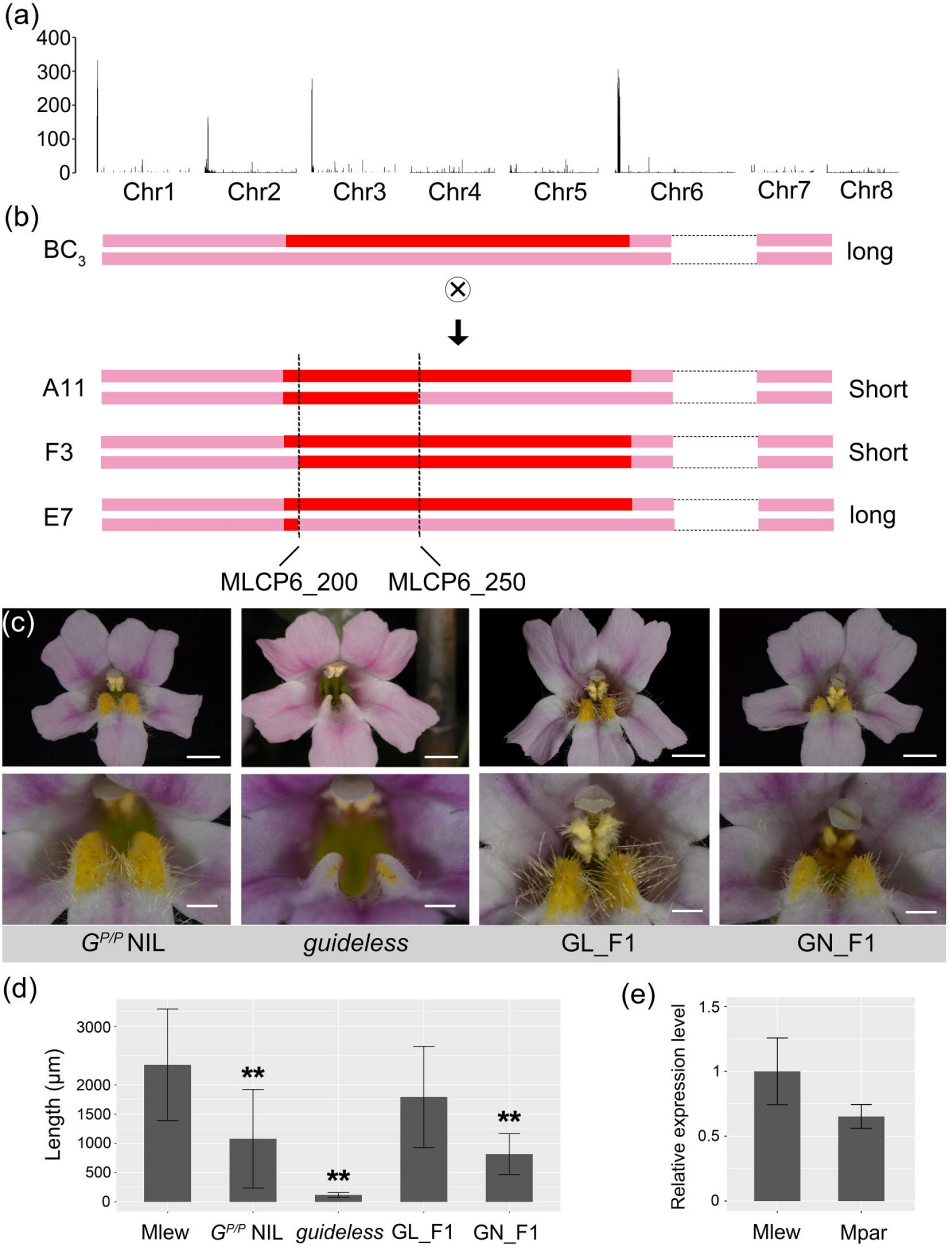
Identification of the causal gene *GUIDELESS*. (**a**) Genome scan of the *G*^*P/P*^ NIL for regions that are enriched in homozygous SNPs compared with the *M. lewisii* reference genome. (**b**) Cross design to generate a fine-scale mapping population (BC_3_S_1_), and within this population, the three most informative recombinants reduced the candidate genomic interval to a smaller region (460 Kb) between markers MLCP6_200 and MLCP6_250. Nectar guide trichome length phenotypes (long vs. short) are shown on the right. (**c**) Front view of the whole corolla and enlarged view of the corolla throat of the *G*^*P/P*^ NIL, the *guideless* mutant, F1 between *guideless* and the wild-type *M*. *lewisii* (GL_F1), and F1 between *guideless* and the *G*^*P/P*^ NIL (GN_F1). Scale bars, 5 mm (top row) and 2 mm (bottom row). (**d**) Quantification of nectar guide trichome lengths (*n* = 20 for each genotype). Error bars are 1 SD. Asterisks indicate differences from the wild-type *M*. *lewisii* (** *p* < 0.01, student’s t test). (**e**) Expression analysis of *GUIDELESS* by RT-qPCR

To identify the chromosomal location of this locus, we performed a bulked segregant analysis of pooled DNA samples from 39 short-haired individuals in the BC_2_S_1_ population. Illumina sequencing of the bulked DNA revealed four relatively small genomic regions homozygous for *M*. *parishii* (Fig. 2a), one of which was expected to contain the causal gene. From the BC_2_S_1_ population, we selected one short-haired individual that was most similar to *M. lewisii* and backcrossed it one more time to *M. lewisii*. Selfing one of the BC_3_ individuals produced the BC_3_S_1_ population. By fine-mapping 96 BC_3_S_1_ individuals, we found that a 460-kb DNA segment on chromosome 6 (Chr 6: 1,533,002–1,988,221; between markers MLCP6_200 and MLCP6_250) co-segregated with nectar guide trichome length (Fig. 2b). This segment contains the *GUIDELESS* gene, which encodes a MIXTA-like R2R3-MYB transcription factor and was previously shown to control nectar guide trichome development in *M*. *lewisii* through mutant analysis [46]. Therefore, *GUIDELESS* was considered the most promising candidate gene. Genotyping the BC_3_S_1_ population also allowed us to identify a high-quality NIL that is homozygous for the *M*. *parishii GUIDELESS* allele in an otherwise *M*. *lewisii* genomic background, named the *G*^*P/P*^ NIL (Fig. 2c).

### Verification of *GUIDELESS* as the causal gene

To verify gene causality, we performed a complementation test by crossing the previously characterized loss-of-function *guideless* mutant (in the *M*. *lewisii* LF10 background; Fig. 2c) with the *G*^*P/P*^ NIL. If *GUIDELESS* is the causal gene underlying the short trichome phenotype of the *G*^*P/P*^ NIL, we would expect that crossing the null *guideless* mutant with the *G*^*P/P*^ NIL, which is homozygous for the recessive *M*. *parishii* allele, should result in F1 progeny (GN_F1) with short trichomes (i.e., the recessive *G*^*P*^ allele and the null mutant allele do not complement one another). This was exactly what we observed: the F1 progeny (GN_F1) produced nectar guide trichomes of comparable length to those in the *G*^*P/P*^ NIL (Fig. 2c, d). By contrast, the F1 progeny from the cross between the *guideless* mutant and wild-type *M. lewisii* (GL_F1) produced nectar guide trichomes comparable to the wild type in length (Fig. 2c, d). These results provide strong evidence that *GUIDELESS* is indeed the causal gene underlying the *G*^*P/P*^ NIL phenotype.

The observation that nectar guide trichomes in the *G*^*P/P*^ NIL are much shorter than those in the wild-type *M*. *lewisii* but still substantially longer than those in the null *guideless* mutant (Fig. 2d) indicates that the *GUIDELESS* allele in *M*. *parishii* is hypomorphic (i.e., partially functional).

**Fig. 3:**
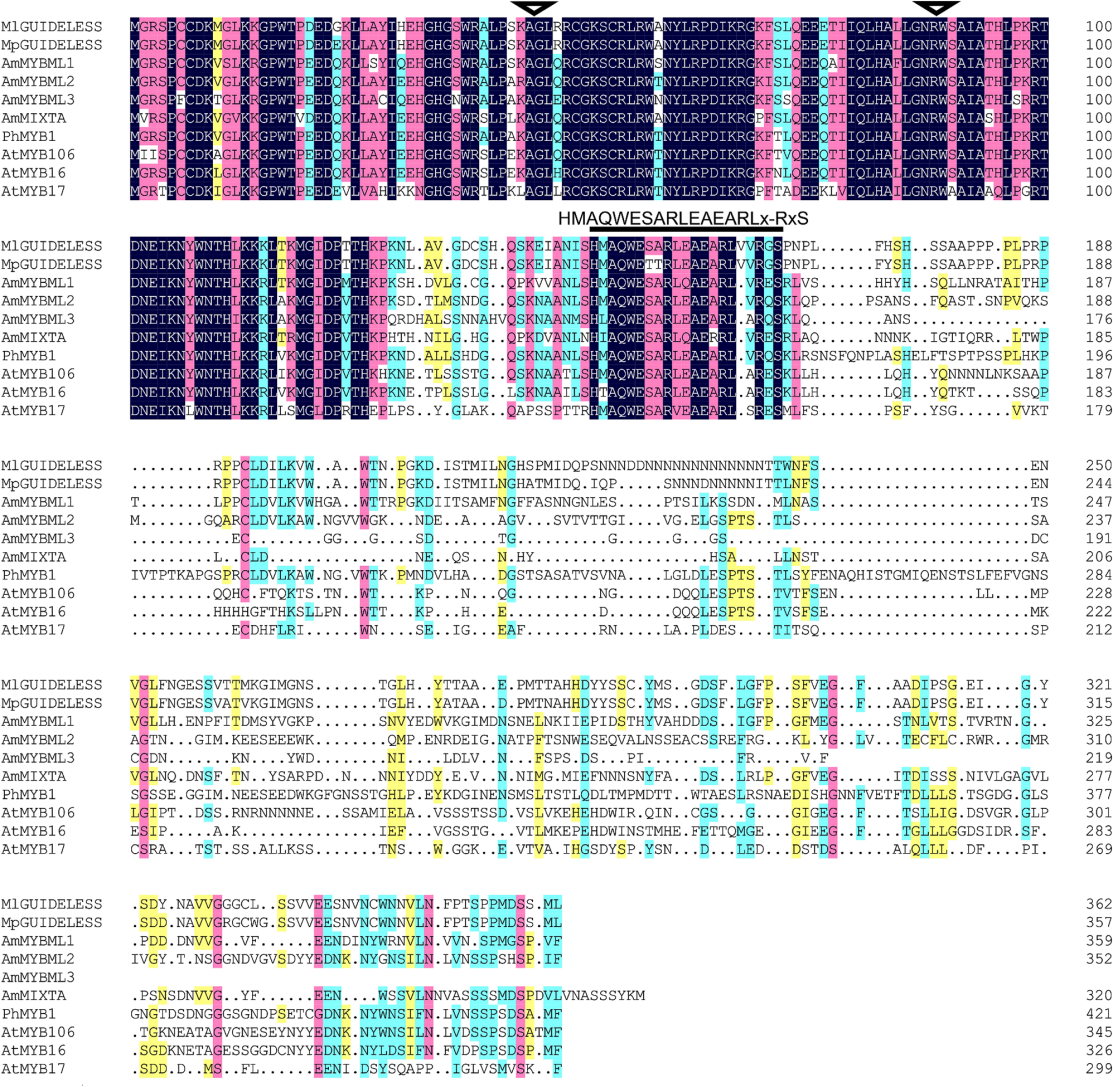
Multiple alignment of MIXTA-like R2R3-MYB protein sequences. Ml: *Mimulus lewisii*; Mp: *Mimulus parishii*; Am: *Antirrhinum majus*; Ph: *Petunia hybrida*; At: *Arabidopsis thaliana*. The signature motif defining the MIXTA-like clade of R2R3-MYBs is marked by a black bar above the alignment. MpGUIDELESS has the two amino acids “SA” in the highly conserved signature motif replaced by “TT”. The two black triangles show the positions of the two introns

Consistent with this inference, expression level of the *MpGUIDELESS* allele was only slightly lower than that of the *MlGUIDELESS* allele (Fig. 2e) and the *MpGUIDELESS* coding DNA does not contain obvious null mutations (e.g., premature stop codons). However, the predicted MpGUIDELESS protein sequence has two amino acid substitutions (“SA” to “TT”) in a highly conserved motif among MIXTA-like R2R3-MYBs (Fig. 3), potentially attenuating protein function.

### Other loci underlying nectar guide trichome length variation between *M. lewisii* and *M. parishii*

Our measurements showed that the *GUIDELESS* locus only accounts for ~ 40% of the parental difference in nectar guide trichome length between *M. lewisii* and *M. parishii* (Fig. 2d), implying the involvement of other loci underlying trichome length variation. Moreover, the fact that F1 hybrids between *M*. *lewisii* and *M*. *parishii* have much shorter nectar guide trichomes than *M*. *lewisii* (Fig. 1a, c, d), whereas the *GUIDELESS* NIL is similar to wild-type *M*. *lewisii* in a heterozygous state, suggests the existence of at least one other locus negatively regulating nectar guide trichome length, with the *M*. *parishii* allele dominant over the *M*. *lewisii* allele. In fact, the proportion of F2 individuals with very short trichomes (class I: 14.4%) is remarkably consistent with the expected ratio of a three-loci model. If the *M*. *parishii* allele is also dominant at the third locus, individuals with very short trichomes are expected to be homozygous for the *M*. *parishii* allele at *GUIDELESS*, and either homozygous for the *M*. *parishii* allele or heterozygous at the other two loci. That is, a ratio of 14.1% (1/4 * 3/4 * 3/4 = 9/64).

## Discussion

In this study we analyzed the genetic basis of variation in nectar guide trichome length between two *Mimulus* species with distinct pollination syndromes. The two species differ by 8-fold in nectar guide trichome length. Our results suggest that the *GUIDELESS* gene is a major contributor to nectar guide trichome length variation between the bumblebee-pollinated *M. lewisii* and self-pollinated *M. parishii*. Compared to other pollinator-associated floral traits such as coloration, nectar guide trichome variation has received little attention. Our study shows that in the *M*. *lewisii*-*M. parishii* system, nectar guide trichome is a typical “complex” trait, as reflected by the continuous variation in the F2 population, but can be dissected into just a few mendelian loci by constructing NILs.

The level of difficulty in generating high quality NILs (e.g., with introgressed chromosome fragments less than 500 kb) and ultimately identifying the causal genes depends on local recombination rates. The *GUIDELESS* locus has an exceptionally high recombination rate. Genotyping 96 ZH101_BC_3_S_1_ individuals was sufficient to narrow down the causal gene to a 460-kb interval (Fig. 2b). The high recombination rate in this region is also consistent with previous bulked segregant analysis of the *guideless* mutant [46]. Genome sequencing of 100 individuals from an intra-specific F2 population was enough to locate the causal mutation to a 50-kb interval. However, it is not unusual for certain loci to undergo low recombination in inter-specific crosses. For example, in our previous studies of flower color variation between *M. lewisii* and the closely related *M. cardinalis*, as well as between *M. parishii* and *M. cardinalis*, the generation of high quality NILs of flower color loci required genotyping of 1,000–3,000 individuals from the final mapping population [61–64]. Generation of high quality NILs at the other inferred nectar guide trichome loci is currently underway. The anticipated NILs will enable us to investigate the genetic interactions among these loci and to ultimately identify the causal genes.

The most recent phylogenomic analysis of *M*. *lewisii*, *M*. *parishii*, and their close relatives [60] suggests that the self-pollination syndrome of *M*. *parishii* is a derived state, including not only short necatar guide trichomes, but also small flower size, inconspicuous coloration, and reduced anther-stigma separation. The inconspicuous coloration of *M*. *parishii* flowers was recently shown to be caused by a mutation in the 5’ UTR of an anthocyanin-activating *R2R3-MYB* gene *PELAN* [61]. This mutation does not alter protein function or gene transcript level. Instead, it inhibits protein translation, and therefore represents a type of loss-of-function mutation underlying the recessive allele in *M*. *parishii*. Although the specific molecular mechanisms are very different, the *GUIDELESS* allele in *M*. *parishii* is somewhat similar to the *PELAN* case, in that it is also recessive and probably encodes a protein with attenuated function. Similar loss-of-function mutations resulting in decreased trichome formation have also been observed in cucumber, wherein the C2H2 transcription factor Tu is entirely absent, leading to the manifestation of the non-warty fruit trait [55].

By contrast, at the other nectar guide trichome loci, the *M*. *parishii* alleles are dominant and, based on the F1 phenotype (Fig. 1a, c, d), most likely encode repressors of trichome elongation. Although transgenic experiments showed that B-type cyclin-like and Jasmonate ZIM (JAZ) proteins can repress trichome initiation in systems other than *Arabidopsis* [51, 56–58], whether these repressors contribute to natural variation in trichome development is unclear. Interestingly, a recent study investigating natural trichome variation in vegetative tissues among snapdragon (*Antirrhinum*) species identified a glutaredoxin gene as a dominant repressor of trichome development [65]. However, this glutaredoxin gene has acquired its trichome repressing function after the divergence between *Antirrhinum* and *Mimulus* [65], and thus is unlikely to cause trichome variation between our focal *Mimulus* species. Moreover, compared to loss-of-function mutations, the molecular mechanisms generating gain-of-function mutations are much less understood. Further characterization of the dominant repressor loci in *M*. *parishii* will not only be significant for the elucidation of negative regulators of trichome development, but also contribute to our understanding of the molecular basis of gain-of-function mutations during phenotypic evolution.

## Conclusions

In this study, we performed the first genetic analysis of the variation in nectar guide trichome length between the bumblebee-pollinated *M. lewisii* and self-pollinated *M. parishii*. The frequency distribution of F2 phenotypes implies a non-monogenic basis for the variation. Through NIL construction, bulked segregant analysis, fine-scale genetic mapping, and complementation cross, we found that the *MIXTA*-like *R2R3-MYB* gene, *GUIDELESS*, is a major contributor to the nectar guide trichome variation between the two species. Furthermore, we inferred that besides *GUIDELESS*, at least one other locus encoding repressors of trichome elongation is also responsible for the short trichome length in *M. parishii*. These results suggest that during a pollination syndrome switch, changes in even seemingly complex traits such as nectar guide trichomes could have a relatively simple genetic basis, and that NIL construction followed by fine-scale genetic mapping is a powerful approach to dissect the genetic basis of these evolutionary transitions.

### Electronic supplementary material

Below is the link to the electronic supplementary material.

Supplementary Material 1

## Data Availability

The Illumina sequencing data of 39 short-haired individuals have been deposited to NCBI under the accession number PRJNA1021984. The raw datasets have been deposited to the Dryad Digital Repository: https://datadryad.org/stash/share/7c-E3lKh-zfdObRqsX37XAIWPwilBAitvpmzP3VNP_E (doi:10.5061/dryad.34tmpg4rm) [66]. Supplementary material is available online.
