# Supplementary material for: Genetic basis of nectar guide trichome variation between bumblebee- and self-pollinated monkeyflowers (Mimulus): role of the MIXTA-like gene GUIDELESS
Source: BMC Plant Biol. 2024 Jan 23;24:62. doi: 10.1186/s12870-024-04736-y (PMC10804488; doi:10.1186/s12870-024-04736-y)
Supplement: Supplementary file 1 — Supplementary Material 1 [file 12870_2024_4736_MOESM1_ESM.docx]

**Table S1** Sequences of primers used in this study.

| **Primer** | **Sequence (5’-3’)** | **Usage** |
| --- | --- | --- |
| Guideless_SP3F | TAGCCGTTGGTGATTGCAGCCA | RT-PCR |
| Guideless_SP3R | GCCCACACTTTGAGTATGTCCA | RT-PCR |
| *MIUBC*_F | GGCTTGGACTCTGCAGTCTGT | RT-PCR |
| *MIUBC*_R | TCTTCGGCATGGCAGCAAGTC | RT-PCR |
